# Activation in a Frontoparietal Cortical Network Underlies Individual Differences in the Performance of an Embedded Figures Task

**DOI:** 10.1371/journal.pone.0020742

**Published:** 2011-07-20

**Authors:** Elizabeth Walter, Paul Dassonville

**Affiliations:** Department of Psychology and Institute of Neuroscience, University of Oregon, Eugene, Oregon, United States of America; University of Granada, Spain

## Abstract

The Embedded Figures Test (EFT) requires observers to search for a simple geometric shape hidden inside a more complex figure. Surprisingly, performance in the EFT is negatively correlated with susceptibility to illusions of spatial orientation, such as the Roelofs effect. Using fMRI, we previously demonstrated that regions in parietal cortex are involved in the contextual processing associated with the Roelofs task. In the present study, we found that similar parietal regions (superior parietal cortex and precuneus) were more active during the EFT than during a simple matching task. Importantly, these parietal activations overlapped with regions found to be involved during contextual processing in the Roelofs illusion. Additional parietal and frontal areas, in the right hemisphere, showed strong correlations between brain activity and behavioral performance during the search task. We propose that the posterior parietal regions are necessary for processing contextual information across many different, but related visuospatial tasks, with additional parietal and frontal regions serving to coordinate this processing in participants proficient in the task.

## Introduction

**Figure 1:**
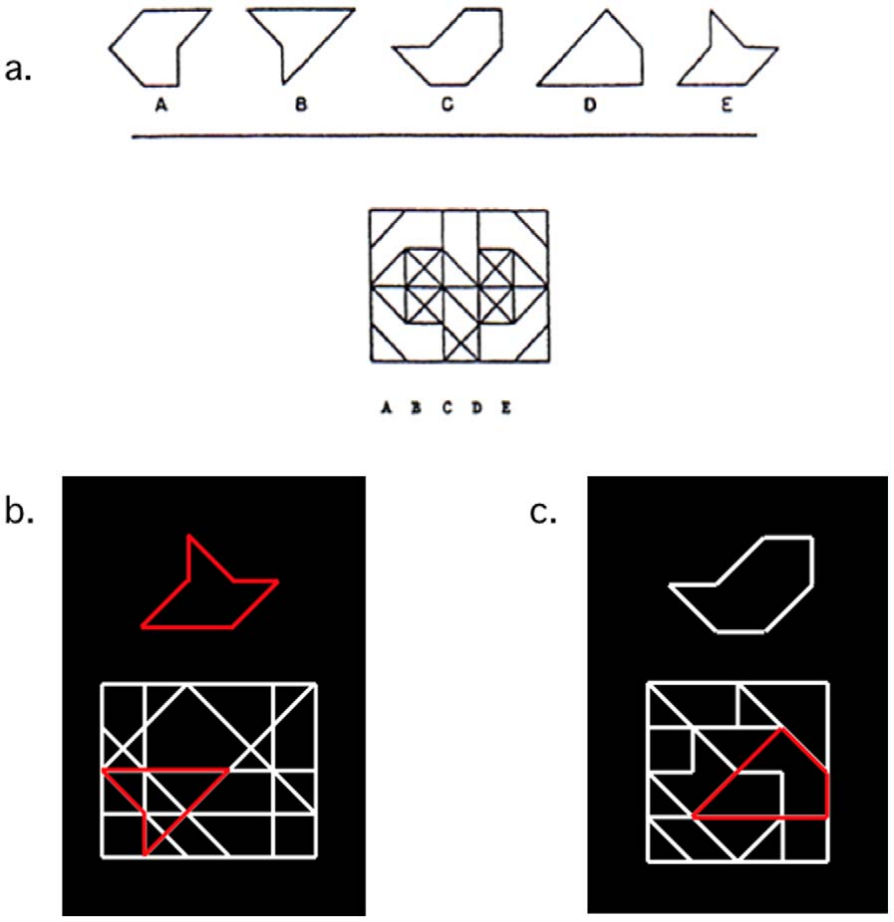
The Embedded Figures Task (and related variant) measures a participant's ability to locate a simple shape within a complex figure. (a) An example of the original Hidden Figures Task. Participants are asked to determine which of the five simple shapes is hidden in the complex shape below (from Ekstrom et al., 1976). Stimuli of the present study (b,c) comprised two potential tasks. When the top shape was red (b), the participant determined whether the simple shape matched the (red) pop-out stimulus in the lower figure (*matching* task). When the top shape was white (c), the participant judged whether the simple shape was hidden inside the complex image below (*search* task). In the stimulus shown, the simple shape was included in the complex image (the lower left slanted line of the complex image forms the lower left slanted line of the simple shape).

**Figure 2:**
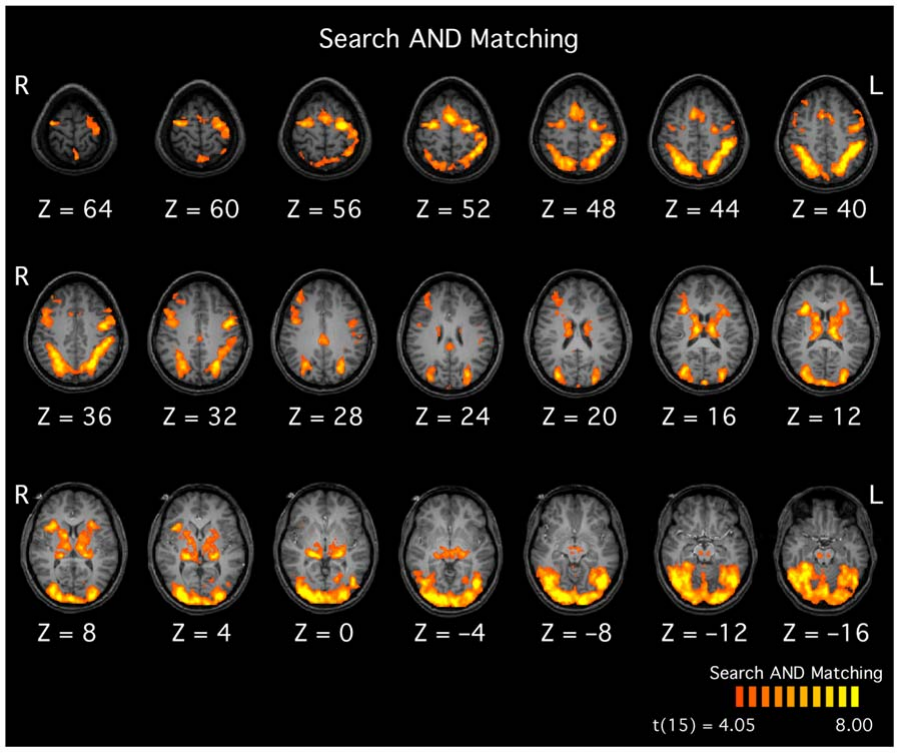
Functional maps demonstrating widespread regions in occipital, temporal and parietal cortex that were significantly active during both the search and matching trials. All activations surpassed a whole-brain voxel-level threshold of *p*<0.001 (uncorrected) with a cluster-corrected threshold of *p*<0.05.

In an increasingly chaotic visual environment, we are often challenged to find a particular object hidden among distracting items. In a typical day, we might search for a bike in a packed bike rack, look for a particular paper or book on a cluttered desk, or try to find an important news item on a disorderly webpage. In order to successfully complete these tasks, we must suppress the extraneous, surrounding items in order to focus on the target object. Researchers have studied this ability with a laboratory exercise known as the Embedded Figures Test (EFT, [1]) or with variants such as the Hidden Figures task (HFT, Figure 1a; [2]). In each of these tasks, participants must determine whether a simple geometric shape is embedded inside a more complex figure composed of many intersecting lines. (Because of the overarching similarity between the Embedded and Hidden Figures Tasks, we will not distinguish them further; the term EFT will be used to refer to both, unless otherwise specified).

As one might imagine, there are large individual differences in the ability to perceptually “disembed” the hidden figure in the EFT. Interestingly, Witkin and colleagues [3] noted an inverse correlation between EFT performance and susceptibility to an illusion of context – the rod-and-frame illusion, in which a vertical rod is seen as slightly tilted when viewed in the presence of a surrounding frame tilted at an angle away from vertical [4]. That is, individuals who excel in the EFT tend to be less affected by the presence of the tilted frame in the rod-and-frame illusion. This series of results led Witkin and colleagues to construct a theory of cognitive processing style, which he termed field dependence/independence (FDI) [5]. In general, field-*dependent* individuals seem to be more affected by the contextual information present in a situation (or visual image) and cannot easily disembed parts from the whole. Field-*independent* individuals, on the other hand, are better able to focus on the details of the image while ignoring contextual information. This tendency of the latter group would make them better able to suppress the gestalt of the EFT in order to focus on the individual line segments that form the hidden target shape, as well as to ignore the misleading, tilted frame in the rod-and-frame task. Work in our lab (Dassonville, Walter, and Bochsler, 2007, presented at the Annual Meeting of the Vision Sciences Society) has recently demonstrated that performance in the EFT is also negatively correlated with susceptibility to another contextual visual illusion, the induced Roelofs effect (in which the perceived location of a target is biased to the left or right when presented in the context of a large illuminated frame that is offset right or left of the observer's midsagittal plane, respectively; [6], [7], [8]). These tasks appear to be linked by virtue of the need to suppress contextual information in order to perform optimally (i.e., to score well on the EFT and to be immune to the Roelofs and rod-and-frame illusions).

Though these tasks are linked theoretically and correlated behaviorally, the neural mechanisms underlying visuospatial contextual processing remain unclear. A previous imaging study performed in our laboratory found bilateral regions of posterior parietal cortex to be selectively active when the stimuli included visuospatial contextual information that led to the induced Roelofs effect [9]. Because susceptibility to the Roelofs effect and performance in the EFT are correlated behaviorally, we surmised that similar neural structures might be involved in processing the visuospatial contextual information in the two tasks. Indeed, other researchers have reported similar parietal regions of activation when participants performed a variant of the EFT [10], [11], [12]. However, it is impossible to determine whether identical posterior parietal areas are involved in processing visuospatial contextual information in the two tasks, given the differences in task methodology and scan parameters across studies and differences in brain anatomy across participants.

The main objectives of the current study are two-fold. First, we examine the neural basis of the disembedding process associated with the EFT, with the particular goal of gaining an understanding of the individual differences in task performances associated with field dependence/independence. Second, we directly test the hypothesis that identical neural areas are recruited for processing the visuospatial contextual information that leads to the induced Roelofs effect and degraded performance in the EFT. This was accomplished by inviting participants who previously participated in our Roelofs imaging study [9] to return and perform a variant of the EFT in the same scanner, using identical scanning parameters. The existence of overlapping regions that are activated in the two tasks would suggest a common substrate for the processing of the contextual information relevant to field dependence in a wide array of tasks. Alternately, entirely independent regions of activation in the two tasks would suggest that although our conceptual use of the term “visuospatial context” has overlapping cognitive theoretical implications, it is divisible neurologically into more specialized processing networks.

## Materials and Methods

### Participants

Experimental procedures were approved by the Institutional Review Board at the University of Oregon, and informed, written consent was obtained from each participant. Sixteen right-handed participants (12 female; 18–28 years of age) were compensated either with money or course credit for an introductory psychology course. Most participants (n = 10) in the current study had participated in our earlier study of the Roelofs effect [9], however all remained naïve to the overall goals of the current study. In addition, nine of the participants completed a localizer task designed to highlight areas of the brain involved in making eye movements. The EFT scanning session lasted approximately 1–1.5 hours.

### Behavioral tasks

Participants performed a variant of the EFT [1] during this event-related functional imaging study. All images were presented to participants on a screen that was viewed via an angled mirror attached to the head-coil. Participants indicated their response via an MR-compatible custom-built keypad, using the index and middle finger on the right hand. During each trial, participants viewed a black background with a simple shape (drawn in white or red; approximately 3° by 5° visual angle) presented simultaneously above a more complex lined figure (drawn predominately in white with a subset of the lines, comprising a closed shape, drawn in red, all spanning approximately 7° by 7°; see Figure 1b and c). The color of the simple shape determined the task to be performed. When the simple shape was drawn in red (Figure 1b), the participant was to perform a shape *matching task*, indicating via button press whether the simple shape matched the red form that “popped-out” of the complex figure below (a button-press by the index-finger indicated that the two red shapes matched, while a press by the middle-finger indicated no match). If the simple shape was instead drawn in white (Figure 1c), the participant was asked to determine whether that shape was hidden in the complex figure below (*search task*; a button-press by the index-finger indicated that the simple shape was hidden in the complex figure, while a press by the middle-finger indicated an absence of the simple shape within the complex figure). When the simple shape was contained within the complex figure, it was always the same size and orientation as the simple shape presented above. In half of the matching trials, the red “pop-out” shape matched the simple shape, while in the other half it did not. Similarly, half of the search trials contained a hidden figure and half did not. The stimuli remained on the screen until a response was made, at which point the screen went blank for a pre-specified intertrial interval (ITI, 1–16 s) before the next stimuli were presented. If no response was made, the trial timed out after 12 seconds, and the next trial began after the ITI.

For the match trials, we instructed our participants to respond as soon as they had determined whether the items matched or not, but emphasized that accuracy was more important to us than speed. For the search trials, participants were asked to respond as soon as they found the simple shape hidden inside the complex figure, and were asked to indicate that the shape was absent only after they were fairly certain it was absent. If the participants were not sure if the target shape was present or absent, they were to keep looking until they could make a decision, or until the trial timed out (after 12 seconds).

Participants performed forty trials (20 matching and 20 search) during each run, which lasted approximately 6.5 min (run durations varied somewhat across participants, depending on the individual differences in response time). Participants each performed six runs (for a total of 120 matching and 120 search trials) over the course of the experiment. A different complex figure was used for each of the 240 trials, in order to prevent any explicit or implicit learning that might otherwise occur if the stimuli were reused. Thirty-two of the stimuli were modifications of those used in the standard HFT [2], while the remaining 208 were custom-made to match the style, size and complexity of those used in the HFT. Each trial used one of five simple shapes, which had an equal probability of being associated with a “present” or “absent” correct response. Trials were subsequently analyzed with respect to whether the participant answered correctly or not (or did not respond). Thus, a trial could end up as one of twelve types (see Table 1), depending on the task (matching or search), response (“present”, “absent” or timed-out) and evaluation of the response (correct or incorrect).

**Table 1:** Proportion (mean ± s.d., %) and response times (ms) of the different stimulus-response types in the matching and search tasks.

		Participant's Response					
Task	Target	“Present”		“Absent”		None	
		%	RT (ms)	%	RT (ms)	%	RT (ms)
**Matching**	**Present**	97.6±0.1	1399±314	0.8±0.1	1008±252	1.6±0.1	12000*
	**Absent**	0.5±0.1	4016±3739	98.0±0.3	1599±627	1.5±0.3	12000*
**Search**	**Present**	76.9±0.7	4591±943	13.5±0.5	7596±2127	9.6±0.4	12000*
	**Absent**	3.9±0.2	6015±2532	73.6±1.5	8352±1559	22.5±1.4	12000*

*Trials timed out after 12 s if no response was made.

### Eye movement localizer task

Because we wanted the participants to view the EFT stimuli in a natural manner, we did not restrict their eye movements while in the scanner. However, it is likely that the search task required more eye movements than did the matching task (as the searching task was generally more difficult and took longer to perform). In order to account for functional activations possibly caused by differences in the patterns of eye movements across the two tasks, an eye movement localizer task was carried out with a subset of participants. In an additional run, participants (n = 9) made eye movements to small targets (0.5°×0.5°, 1 s duration each, no ISI) that appeared on a black background, during 20 s blocks separated by intervening periods of rest (16 s). Eye-movement blocks were preceded by 2 s of the instruction “Follow target.” Participants were instructed to foveate each target during the movement blocks, and lay still with their eyes open, but without making eye movements, during the rest periods.

### Scan parameters and image processing

Functional MR images were acquired using a 3T head-only MRI (Siemens Magnetom Vision, Erlangen, Germany), with a standard birdcage head-coil. For each functional run, we used a standard BOLD (blood oxygenation level dependent) gradient-echo EPI (echo-planar imaging) sequence (TR = 2 s, TE = 30 ms, 32 slices, 4 mm thickness, 0 mm gap, FOV = 200×200×128 mm) allowing us to achieve nearly whole-brain coverage with an in-plane resolution of 3.125×3.125×4 mm. In addition, we collected anatomical images (whole brain, 1 mm slices, 0 mm gap) using a standard MPRAGE sequence, yielding an in-plane anatomical resolution of 1×1×1 mm. To ensure that the laterality of the images would be correctly interpreted, participants were scanned with a small marker (0.5 ml centrifuge tube filled with a nickel sulfate solution) taped to the right side of the forehead.

Stimulus presentation durations in this event-related design were dependent on the rate of each participant's response, while ITIs ranged from 1–16 s in a manner designed to maximize our ability to reconstruct the hemodynamic response signal for each study condition (see [13]). We used the optseq program (http://surfer.nmr.mgh.harvard.edu/optseq/) to yield near-optimal sequences of trial types (search or matching task, target shape present or absent) and ITIs for each run, for each participant [14]. Though we attempted to optimize the sequence of trial types and ITIs, the variability in participant response time and response choice made it impossible to precisely determine the optimal stimulus sequence in advance.

Raw functional images from each participant were converted into BrainVoyager format and pre-processed using custom MatLab scripts to automate portions of the processing and to ensure standardization across all runs. Images were preprocessed with a slice-time scan correction, 3-D motion correction, a temporal high-pass filter to remove the 1^st^, 2^nd^ and 3^rd^ order elements, and a spatial low-pass filter that smoothed the data using a 4 mm FWHM Gaussian smoothing kernel. Finally, the functional images from each participant were aligned to their own anatomical scan, which were then aligned with the AC-PC plane and converted into the Talairach atlas space as defined by Talairach and Tournoux [15] by individually defining bounding boxes for the entire brain, using AC and PC set as anchor points for the transformation (BrainVoyagerQX 1.9).

Data from all participants were combined and analyzed using the general linear model with separate predictors for matching and search trials. For the predictors of both types, error trials (i,e,, those in which the participants answered “present” when the target was absent from the complex figure, or vice-versa) were discarded from analyses and not considered further. The predictor for the search trials, however, also included timed-out trials in which the target shape was absent from the complex figure; although these trials were not technically “correct”, it was assumed that the absence of a response indicated that participants maintained their search for the hidden figures up until the time at which the trial timed-out. Since the focus of the study was on the disembedding process that was being attempted during this search process, it was deemed appropriate to include these timed-out trials in the analyses. We used a duration of 12 s (the maximum duration of any trial) for these time-out trials.

The predictors, delineating the duration of the individual trials of a given type from stimulus presentation until response/time-out, were convolved with the hemodynamic response as implemented in the BrainVoyagerQX 1.9 statistical package. Below, we first present the results of a random-effects analysis of all sixteen participants (using a voxel-wise threshold of *p*<0.001 uncorrected, with a cluster-correction threshold of *p*<0.05; cluster-based corrections were performed using the Cluster-level Statistical Threshold Estimator plug-in for BrainVoyagerQX 1.9, [16]). In addition, we present the results of a fixed effects analysis (voxel-wise threshold of *p*<0.05, Bonferroni corrected) of the ten participants who took part in both the present EFT study and our imaging study of the Roelofs effect [9].

## Results

### Behavioral results

Although participants responded correctly on the majority of trials, there were significant differences in the error rates and response times in the search and matching tasks (error rate_matching_ = 0.7±1.1% (mean ± s.d.), error rate_search_ = 12.0±6.8%, *t*(15) = 6.77, *p*<0.001; response time_matching_ = 1637±629 ms, response time_search_ = 7199±1621, *t*(15) = 13.89, *p*<0.001; see Table 1 for a full description of these performance measures). Because each participant correctly answered a different number of trials, and did so with varying response times, we quantified each participant's overall performance in the tasks using the methods of information theory [17]. With the two-alternative decisions required in the present tasks, each response could transmit, at most, one bit of information, with errors decreasing the average information to less than one bit per trial. Dividing this value by the average response time for each participant yielded a measure of performance (processing speed, expressed in bits/s) that is less prone (compared to the separate measures of accuracy and response time) to the difficulty that is introduced by individual differences in a speed-accuracy tradeoff. On average, the matching trials elicited processing speeds of 0.62±0.19 bits/s, while the search trials had significantly slower processing speeds of only 0.076±0.041 bits/s (*t*(15) = 11.80, *p*<0.001).

### Neural activations associated with both tasks

The results of the random-effects analysis demonstrated that extensive cortical and subcortical activations were associated with both the matching and search tasks (Figure 2). In the cortex, prominent bilateral activations were seen in much of the occipital lobe and inferotemporal cortex, along the intraparietal sulcus, and in the anterior cingulate and middle frontal gyri. Subcortically, activations were seen in the basal ganglia, thalamus and midbrain. Because of the difficulty in parsing these large regions of activation, and because the goal of the study was to focus on the activations that distinguished the matching and search tasks, no attempt was made to delineate these activations further.

### Neural activations associated with the matching task

**Figure 3:**
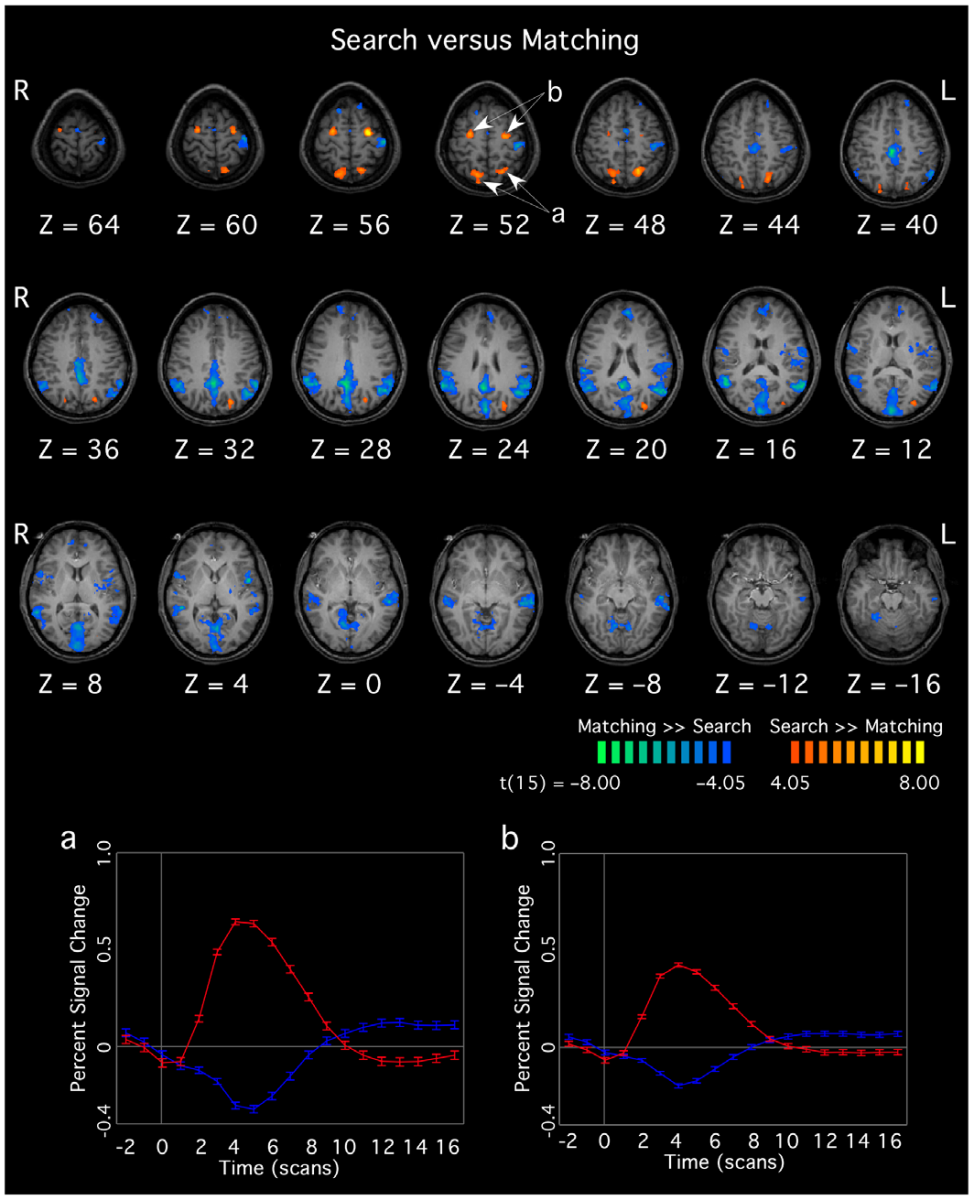
Functional maps demonstrating regions that were more active during the search (warm colors) or matching trials (cool colors). The event-related averages (bottom panels, corresponding to the labeled arrows in the brain images) show the time course of the activation from the search (red) and matching trials (blue) in the search-specific regions of parietal and frontal cortices. All activations surpassed a whole-brain voxel-level threshold of *p*<0.001 (uncorrected) with a cluster-corrected threshold of *p*<0.05.

A contrast of the correct matching trials versus the correct (and timed-out) search trials indicated several cortical regions whose activations more closely fit the time course of the predictor for the matching trials (Table 2 and Figure 3, cool colors). These included bilateral activations in the superior and medial frontal, precentral, cingulate and superior temporal gyri, and the cuneus.

**Table 2:** Random-effects analysis for the search versus matching contrast (n = 16).

			Talairach Coordinates				
	BA	Anatomical location	x	y	z	Extent (mm^3^)	average *t*-value
**Matching >> Search**							
Frontal	6	R superior frontal gyrus	16	19	56	273	4.57
Frontal	6	L superior frontal gyrus	−14	25	55	220	4.53
Frontal	9	R superior frontal gyrus	13	53	31	380	4.29
Frontal	8	L superior frontal gyrus	−20	40	39	1013	4.35
Frontal	10	R medial frontal gyrus	9	52	9	229	4.39
Frontal	6	L medial frontal gyrus	−1	−7	51	803	4.51
Frontal	9	L medial frontal gyrus	−6	48	19	2191	4.54
Frontal	44/6	R precentral gyrus	49	7	7	854	4.52
Frontal	43	R precentral gyrus	56	−5	13	977	4.48
Frontal/Parietal	1/2/3/4	L postcentral/precentral gyrus	−41	−27	55	3477	4.72
Occipital	17/18/23/30	R/L cuneus/posterior cingulate	1	−76	12	18793	4.79
Limbic	31/24/29/30	R/L cingulate/posterior cingulate	1	−39	32	11970	5.10
Temporal	13/22/42/39	R superior temporal gyrus	53	−46	20	14087	4.81
Temporal	22	L superior temporal gyrus	−51	−18	3	6901	4.65
Temporal	40	L supramarginal gyrus	−50	−51	23	14595	4.95
Cerebellum		R cerebellum (anterior lobe)	25	−45	−19	1550	4.59
Cerebellum		L cerebellum (anterior lobe)	−36	−49	−22	560	4.54
Cerebellum		R cerebellum (posterior lobe)	22	−73	−37	432	4.52
Cerebellum		L cerebellum (posterior lobe)	−30	−73	−36	262	4.50
**Search >> Matching**							
Parietal	7	L superior parietal	−17	−64	51	2410	4.90
Parietal	7	R precuneus	16	−69	51	2962	4.62
Occipital	31	L precuneus	−23	−76	29	1440	4.68
Frontal	6	R middle frontal gyrus	25	−10	56	1268	4.82
Frontal	6	L middle frontal gyrus	−24	−11	56	1221	5.40

All contrasts were performed using a whole-brain voxel-level threshold of *p*<0.001 (uncorrected) with a cluster-corrected threshold of *p*<0.05. Each entry refers to the center of mass of a single region of activation (reported in the normalized coordinate space of Talairach and Tournoux, 1988), and all Brodmann areas refer to the anatomical region nearest the center coordinate, obtained from the Talairach daemon (Lancaster et al., 2000; http://www.talairach.org/daemon.html).

### Neural activations associated with the EFT

Because the main goal of the this study was to determine the brain areas specifically involved in the disembedding component of the EFT, the functional contrast of greatest interest was a comparison of all correct (and no-response) search trials versus all correct matching trials. This contrast indicated that the EFT was associated with bilateral activations in the parietal lobe (precuneus/SPL, BA 7) and middle frontal gyrus (BA 6), and in left occipital cortex (BA 31; see Table 2 and Figure 3, warm colors).

**Figure 4:**
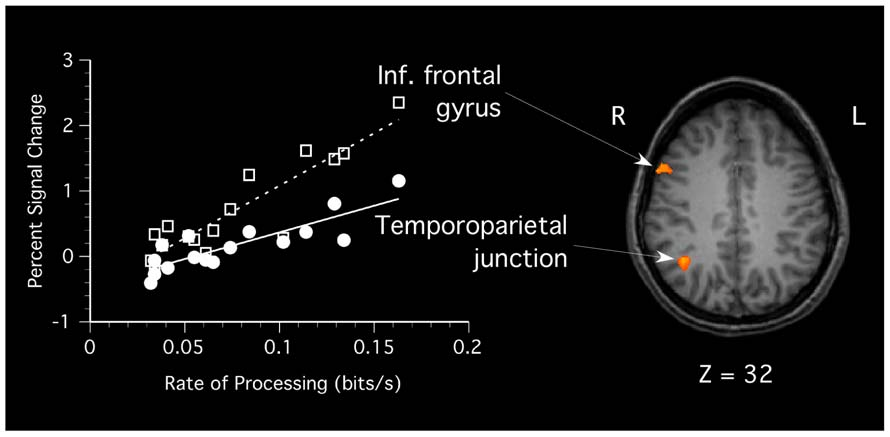
Scatter plot showing the correlation between brain activation and processing speed in the search task (as measured in bits/s) for right-lateralized regions in (a) inferior frontal gyrus (BA 9; center of mass at Talaraich coordinate: 53, 12, 32) and temporoparietal junction (BA 39; 38, −55, 31). All activations surpassed a whole-brain voxel-level threshold of *p*<0.001 (uncorrected) with a cluster-corrected threshold of *p*<0.05, with *R* ^2^ = 0.76 in the inferior frontal gyrus and *R* ^2^ = 0.75 in the temporoparietal junction.

To assess whether the overall speed of processing in the EFT was associated with a differing pattern of neural activity during this task, we performed a whole-brain analysis of covariance (ANCOVA, using a voxel-threshold of *p*<0.001 uncorrected, with a cluster-correction threshold of *p*<0.05), using each participant's speed of processing in the search trials (as measured in bits/s) as a covariate. We found significant correlations between brain activation and processing speed in a predominantly right-lateralized network of frontal and parietal areas. Specifically, we found positive correlations in a portion of right parietal cortex, extending posteriorly from a region in the temporoparietal junction (BA 39; *r* ^2^ = 0.75; see Table 3 and Figure 4) to the precuneus (BA 19; *r* ^2^ = 0.72). In addition to the parietal loci, sections of the right inferior frontal gyrus (BA 9; *r* ^2^ = 0.76) and insula (BA 13; *r* ^2^ = 0.72), and the left precentral gyrus (BA 4; *r* ^2^ = 0.74) showed strong correlations between processing speed and activation. In all of these regions, participants with higher processing speed showed greater activation than did participants with lower processing speed.

**Table 3:** ANCOVA analysis, correlating a measure of processing speed (bits/s) with activation levels in the search trials (n = 16).

			Talairach Coordinates				
	BA	Anatomical location	x	y	z	Extent (mm^3^)	average *R* ^2^
Parietal	19	R precuneus	36	−67	40	415	0.72
Parietal	39	R temporoparietal junction	38	−55	31	636	0.75
Frontal	9	R inferior frontal gyrus	53	12	32	307	0.76
Frontal	4	L precentral gyrus	−31	−26	52	299	0.74
Sub-lobar	13	R insula	45	14	1	148	0.72

All contrasts were performed using a whole-brain voxel-level threshold of *p*<0.001 (uncorrected) with a cluster-corrected threshold of *p*<0.05. Each entry refers to the center of mass of a single region of activation (reported in the normalized coordinate space of Talairach and Tournoux, 1988), and all Brodmann areas refer to the anatomical region nearest the center coordinate, obtained from the Talairach daemon (Lancaster et al., 2000; http://www.talairach.org/daemon.html).

### Neural activation associated with the eye movement localizer task

**Figure 5:**
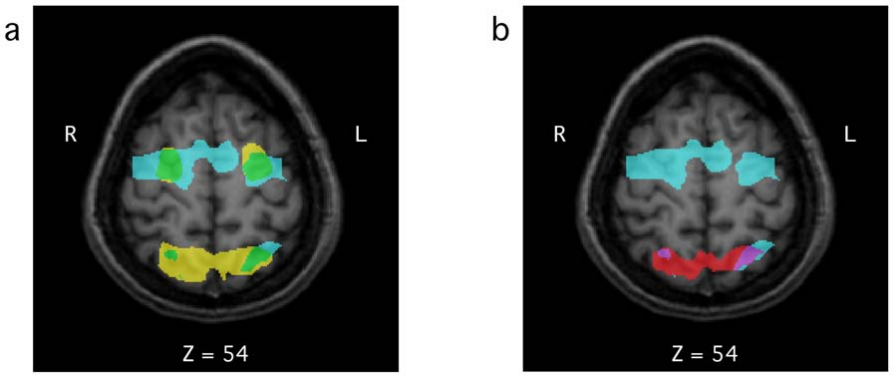
A comparison of the brain activation for performance of the EFT, the Roelofs task, and an eye movement localizer task. (a) Brain regions showing voxels significantly activated in a fixed-effects analysis of all participants who performed an eye movement localizer task, in addition to the EFT (n = 9). Yellow voxels depict those areas significantly more active during the search trials than during the matching trials; blue, regions activated during the eye movement localizer task; and green, regions common to both the search >> masking contrast as well to as the eye movement localizer task. (b) Brain regions showing voxels significantly activated in a fixed-effects analysis of all participants who performed the Roelofs task (Walter and Dassonville, 2008), in addition to the eye movement localizer and EFT. Red voxels depict those areas significantly activated during both the Roelofs task as well as the EFT; blue, regions activated during the eye movement localizer task; and purple, regions common to all three tasks. All contrasts are thresholded at p<0.05, Bonferroni corrected.

To account for possible differential effects of eye movements in the search and matching tasks, we compared our EFT results to those from an additional eye movement localizer task completed by nine of our participants. Because the intent of this analysis was to compare activations within the tested group of participants, we performed a fixed-effects analysis of the eye-movement activations and compared the results with those of a fixed-effects analysis of the EFT data (voxel-wise threshold of p<0.05, Bonferroni corrected). During the eye movement localizer blocks, extensive regions of cortex were significantly activated, including the frontal eye fields (FEF; precentral gyrus) and regions in the parietal cortex, with foci near the intraparietal sulcus. In addition, large regions of the occipital (BA 17/18) and inferior parietal cortices (BA 40) were activated during the eye movement localizer. Although the frontal areas active in the EFT (search >> matching) contrast did overlap with areas implicated in the eye movement localizer task, the profile of parietal activations associated with the EFT was very different from that associated with the eye movement localizer task (see Figure 5a). Indeed, of the parietal cortex activated in the EFT, 76.2% showed no overlap with the areas activated in the eye movement localizer task. In addition, the parietal saccade-related activations did not overlap with the parietal regions whose activations were correlated with individual differences in the rate of processing in the EFT.

## Discussion

The EFT [1] and related paradigms (e.g., [2]) entail a process of searching for and isolating the individual line segments of a geometric shape embedded within the gestalt of a more complex image. It has been demonstrated many times that the ability to perform this disembedding process differs greatly between individuals, a finding that led Witkin et al. [5] to propose this task as a measure of a cognitive construct he called field dependence/independence. From this perspective, field-independent individuals are less affected by the contextual effects of the gestalt, and are better able to isolate the hidden figure. On the other hand, field-dependent individuals are more prone to consider the gestalt, which then is more effective in obscuring the hidden figure and thus hampers search performance.

The tasks employed in the present study were designed in such a way as to isolate the neural mechanisms underlying the disembedding process that defines the EFT. For example, with the exception of the color of the simple shape, the stimuli used in the search task were identical to those of the matching task (e.g., both were of equal size and brightness, had equal levels of complexity, and included a subset of line segments that were differently colored; Figure 1b and c), so that a contrast of the functional activation associated with the two tasks would subtract out the low-level sensory processes that the tasks had in common. Similarly, both tasks required a comparison of geometric shapes, and both required identical motor responses to indicate the presence or absence of the simple shape within the complex image. Thus, the primary difference between these tasks lay in the manner in which the participants disembedded the target shape within the complex image. With the matching task, color was used to cause the target shape to “pop out” from the complex image, whereas the search task could be completed only by a methodical parsing of the line segments contained in the complex image.

A comparison of the activations with the two tasks indicated that this disembedding process is associated with a bilateral frontoparietal network of brain regions, specifically in the superior parietal cortex, precuneus and middle frontal gyrus (Figure 3). In addition to the frontoparietal regions associated with the search task across all participants, the ANCOVA analysis revealed non-overlapping regions that were correlated with the speed of processing in the EFT performance for individual participants. These regions showed a greater activation for participants who were better at the task, suggesting that they may be part of a second processing circuit capable of modulating EFT performance.

The matching >> search contrast showed activations in a surprisingly large number of cortical areas (Figure 3, cool colors), especially given that similar contrasts in previous studies (albeit with paradigms using block designs) showed no areas that were specifically active in the matching task [10], or only a region of left medial temporal lobe [11]. However, in interpreting the matching >> search contrast in the present study, one must consider how this contrast might have been affected by the large differences in response times associated with the matching and search trials, with the matching trials having relatively short and highly consistent response times and the search trials having relatively long and variable response times. For example, given the consistent response times, in the matching trials, the activation of primary motor cortex associated with the button-press report would always be tightly synced to the onset of the trials, whereas it would be delayed by the variable response time in the search trials. Accordingly, the activation of primary motor cortex that can be seen in the matching >> search contrast should not lead to the conclusion that primary motor cortex is more specifically active in the matching task. Similarly, other aspects of the tasks that occur only at the very beginning or end of both trial types (e.g., visual processing and attentional alerting driven by the onset of the stimulus, task switching guided by the color of the simple stimulus, stimulus-response decoding to correctly guide the button-press report) would be expected to be better fit by the short, consistent time course of the predictor for the matching trials. Thus, the regions found to be active in the matching >> search contrast (Figure 3) would reflect not only those areas truly more active in the matching trials, but also those areas that are involved in the processes that are common to both tasks at the beginning or end of the trials. On the other hand, the search >> matching contrast should more specifically reflect the neural mechanisms that are active during the disembedding process associated with the EFT throughout the duration of the search trials.

### Relationship to oculomotor and visual-spatial attention circuitry

The posterior parietal regions implicated in processing visuospatial contextual information are somewhat similar to those regions suggested to comprise a frontoparietal eye-movement and attention network. Although eye movements were not recorded during the present experiment, it can be assumed that the pattern of eye movements differed somewhat between the search and matching tasks. Thus, it is possible that the parietal and frontal activations associated with the EFT were not caused by the disembedding process *per se*, but were rather an artifact of a difference in oculomotor behavior. Indeed, an examination of Figure 5a demonstrates that the frontal regions associated with the EFT did overlap substantially with an eye-movement-related frontal activation (presumably the frontal eye fields) as delineated by the eye movement localizer task. It cannot be ruled out, therefore, that these frontal regions were only active due to a possible difference in the pattern of eye movements associated with the search and matching tasks. In contrast, although the parietal activations associated with the EFT were located near the parietal eye fields, there was in fact very little overlap between these regions of activation. This suggests that the observed parietal activations were not caused directly by a difference in the oculomotor behavior between the search and matching tasks.

The difference between the matching and search tasks of the present study can be conceptualized as a difference between bottom-up (i.e., pop out) processes for locating the target in the matching condition as contrasted with top-down processes mediating search for the embedded figure. Previous work (reviewed by Corbetta and Shulman [18]) has indicated that different neuronal circuits are involved in these two aspects of attentional processing, with regions in temporoparietal and inferior frontal cortices involved in stimulus-driven attention, and regions of the intraparietal, superior parietal and superior frontal cortices involved in the top-down control of the attentional focus. These general patterns of activation are consistent with the activation seen here with the matching and the search tasks, respectively, and, in particular, indicates that the superior parietal cortex plays an important role in the disembedding process associated with the EFT.

Furthermore, it is possible that additional neural circuits come online for those individuals who are more proficient at solving the EFT. Indeed, activation in right-lateralized areas of the temporoparietal junction, precuneus, inferior frontal gyrus and insula is not evident across all participants, but is instead correlated with individual differences in performance on the EFT, with more proficient participants showing greater activation. These areas may be more directly involved in the executive control and coordination of the stimulus-driven and top-down attention required in a search for the hidden figure. In particular, the right inferior frontal gyrus (IFG) has been singled out by other researchers as being critical for inhibitory control [19]. Interestingly, in a task requiring interference suppression, Bunge and colleagues [20] found activation within a network of regions very similar to those found in the correlation analysis in the current study – including right inferior and middle frontal gyri, inferior parietal regions, and insula. Thus, it is possible that, in participants proficient in the EFT, these regions are responsible for inhibiting the distractions of the extraneous line segments in the complex image during the search for the hidden figure. Serences and colleagues [21] also found that a similar network of regions, including right inferior and middle frontal gyri, temporoparietal junction, inferior parietal sulculs, and insula, was involved in coordinating voluntary and stimulus-driven attention in a complex task. In the present study, these regions appeared to be similarly involved in coordinating top-down and bottom-up attention as the participants shifted attention among the components of the complex image in search of the simple shape.

### Comparison with previous studies of the EFT

**Figure 6:**
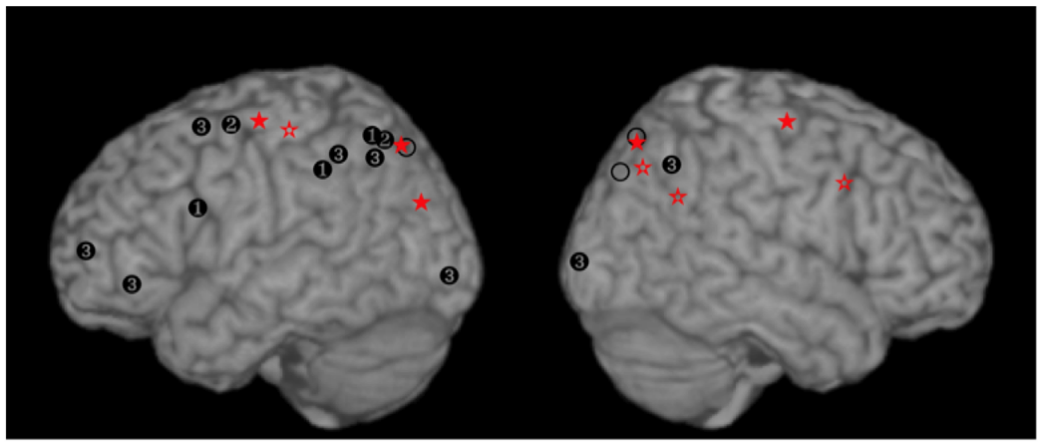
A comparison of the results from the present experiment (*filled stars* indicating the center of mass for significant activations in the search >> matching contrast, Table 2; *unfilled stars* indicating the regions where the activation was correlated with the speed of processing in the search task, Table 3) and those of Manjaly et al. (2003, markers labeled *1*; 2007, markers labeled *2*), and Lee et al. (2007, markers labeled *3*). Only those sites located on the lateral cortical surface are shown; not shown are sites in the superior frontal gyrus/anterior cingulate, left lingual/fusiform gyrus and right parahippocampal/fusiform gyrus (Lee et al., 2007), and a site in the insula whose activation was correlated with the speed of processing in the current study. Finally, the open circles indicate the locations of activations associated with the processing of the illusion-inducing context of the Roelofs effect (Walter and Dassonville, 2008).

Previous imaging studies of typically-developing individuals have also examined the neural activation associated with the disembedding process of the EFT [10], [11], [12]. In general, these earlier studies found a similar network of frontal and parietal areas associated with the task, though with a pattern that was more left-lateralized than that reported here (Figure 6). This difference in lateralization might be attributable to a difference in task difficulty, as evidenced by a rough correlation between the reported lateralization and reaction times in the search tasks of the different studies. Indeed, Manjaly et al. designed relatively simple stimuli so that the accuracy rates could be maintained at more than 85% with a relatively short stimulus duration of 3 s; thus, response times were approximately 2.1 s [10] and 1.8 s ([11], using adolescent participants). These simple hidden figures stimuli were associated with activation only in the left parietal and frontal cortices (locations marked *1* and *2* in Figure 6). Lee at al. [12] used stimuli that evoked longer response times (5.8 s), which led to a bilateral activation in the parietal lobe and a left-lateralized activation in frontal cortex (marked *3* in Figure 6). Mean response times in the EFT of the current study were 7.1 s, and were associated with bilateral parietal and frontal activations (marked with filled stars in Figure 6). This relationship between lateralization and response times suggests that the parietal cortices in a frontoparietal network in the left hemisphere is primarily responsible for the disembedding process with simple stimuli, with an analogous network in the right hemisphere recruited only as the task becomes more difficult.

The present study also went further than previous studies of the EFT in that it used event-related fMRI methods to avoid possible confounds caused by differences in the time-on-task that are inherent in paradigms comparing blocks of trial types with different levels of difficulty. These methods allowed us to examine differences in the patterns of activation that correspond to individual differences in the performance of the task. In particular, we found that participants who were more efficient (that is, who had faster rates of information processing, in bits/s) showed greater levels of activation in the right-lateralized network of frontal and parietal regions (Figure 6, unfilled stars), compared to the less efficient participants. These results suggest that the more efficient performers were those that were more successful at recruiting the resources of the right hemisphere. In the context of field dependence/independence, this suggests that field-independent individuals typically recruit a strongly bilateral network of parietal and frontal regions when performing the EFT. Thus, the difference between field-dependent and field-independent individuals may be the ease at which they can recruit the neural resources of the right hemisphere for this task.

### Relationship to the enhanced EFT performance associated with autism

In addition to the widely-studied individual differences of EFT performance in a typically-developing population, it is also known that EFT performance is affected in individuals with autism. Indeed, individuals with autism excel in the EFT, showing faster, more accurate performance than typically-developing individuals [22], [23], [24], [25]. A recent hypothesis of Baron-Cohen and colleagues suggests that specific autistic traits exist as continua across the general population, with individuals with autism occupying the extremes of these continua (see [26], for a review). Given this, it is interesting to consider the possibility that the boost in EFT performance associated with autism is an extension of the individual differences seen with field-independence in the general population. If this were the case, one would expect individuals with autism to show an extreme version of the pattern of activation seen in the current study with the more efficient EFT performers. Several imaging studies have attempted to assess the neural activations that underlie this enhanced EFT performance within individuals with autism [11], [12], [27] or their parents [28]; unfortunately, results across these studies are highly variable. Ring et al. [27] found that autistic individuals performing the EFT showed more activation in striate and extrastriate regions, and less in premotor and parietal regions, compared to typically developing controls. Manjaly et al. [11] found a similar pattern in general, but only when using lower statistical thresholds and qualitative comparisons between the two groups. Lee et al. [12] also provided only a qualitative comparison of the activations from the two groups, finding that whereas control children had activations in left dorsolateral, medial and dorsal premotor cortex and bilateral activations in parietal, occipital and ventral temporal cortices during the EFT, these were reduced to activations in only left premotor, left superior parietal and right occipital cortices in children with autism. Baron-Cohen et al. [28] showed that the parents of children with autism had decreased activations in the middle occipital and lingual cortices, compared to the parents of typically developing children. However, one should view the results of these previous studies with some skepticism, since each one failed to find a significant difference in EFT performance in their tested groups, perhaps due to stimuli that were too simple, or too few trials. To our knowledge, the current study is the first to demonstrate neural activations that co-vary with individual differences in EFT performance; it is still an open question whether the neural basis for these individual differences reflects a less extreme version of the enhanced EFT performance associated with autism.

### Overlap with contextual processing associated with visuospatial illusions

While the cognitive construct of field dependence/independence has been used as a way of understanding the individual differences in performance of the EFT, it also suggests an explanation for individual differences in illusion susceptibility [3]. This theory suggests that because field-dependent individuals are more prone to taking into consideration the context of a visual scene, they would be more susceptible to context-dependent visuospatial illusions than are field-independent individuals. Witkin et al. [5] originally demonstrated this relationship with the rod-and-frame illusion, showing that individuals that were more prone to the illusion also had a reduced performance (e.g., slower response times) in the EFT. Recent work in our lab has extended these findings to the Roelofs effect, by demonstrating significant correlations between rod-and-frame susceptibility, Roelofs susceptibility and EFT performance [29; see also Dassonville, Walter, and Bochsler, 2007, presented at the Annual Meeting of the Vision Sciences Society].

The behavioral relationship between EFT and illusion susceptibilities suggests a common underlying neural mechanism driving performance in these tasks, and led us to predict that we would find overlapping networks of brain regions involved in processing the contextual information associated with the tasks. Indeed, a previous study of the induced Roelofs effect [9] found regions in parietal cortex that were involved in processing the contextual cue provided by a Roelofs-inducing frame (Figure 6, open circles), and ongoing work in our lab has demonstrated that deactivation of these regions with slow repetitive transcranial magnetic stimulation can decrease susceptibility to the rod-and-frame illusion (Lester and Dassonville, 2010, presented at the Annual Meeting for the Society for Neuroscience). Importantly, these areas also neatly overlap the areas found to be active in the EFT in the present study and those of Manjaly et al. [10], [11] and Lee et al. [12].

However, the present study allows for a more direct test of the hypothesis that similar brain structures are involved in processing the contextual information that leads to greater illusion susceptibility and decreased EFT performance, since 10 of the participants tested in the EFT were also involved in our earlier study of the Roelofs effect [9]. Consistent with previous results, we found a negative correlation (*r* = −0.28) between behavioral performance on the two tasks. Although the correlation in this small sample did not reach significance, it was in the same direction (i.e., participants with a greater susceptibility to the Roelofs illusion had a decreased performance on the EFT) and of similar magnitude to the significant correlations found with larger samples (Dassonville, Walter, and Bochsler, 2007, presented at the Annual Meeting of the Vision Sciences Society). To test for brain regions significantly active in both tasks, we performed fixed effects analyses (thresholded at p<0.05, Bonferroni corrected) of the data in both studies. As we had predicted, bilateral regions of parietal cortex (centered at Talairach coordinates x = 21, y = −71, z = 41 and x = −21, y = −70, z = 39) were active in both tasks (Figure 5b), which suggests that these regions are processing the visuospatial contextual cues that both hinder performance on the EFT and lead to visuospatial illusions such as the Roelofs and rod-and-frame effects. Although the evidence presented here falls short of that required to definitively suggest that these posterior parietal regions are specifically and singularly devoted to processing visuospatial context (see, for example, the inference arguments laid out in [30]), it does suggest a potential neural locus for the correlated reliance on contextual information in these two very different visuospatial tasks.

In this study, we have demonstrated robust activity in parietal and frontal areas during completion of an Embedded Figures Task, while controlling for the non-search aspects of the task (e.g., appearance of the visual figure, presence of a perceptual judgment and button-press response). Whereas the frontal regions of activation may have been attributable to differences in the patterns of eye movements between the search and matching tasks, the spatial pattern of activation seen in parietal cortex was very different from that seen during an eye movement localizer task. Instead, the parietal regions associated with the EFT overlapped in large part with activation found during a very different, but behaviorally related, visuospatial processing task, a location judgment performed within a visuospatial context that leads to the illusion of the Roelofs effect [9]. These results, taken together, suggest that these portions of superior parietal cortex and precuneus are involved in processing the contextual information across a wide range of visuospatial tasks.
